# Association of hypertension and insulin resistance in individuals free of diabetes in the ELSA-Brasil cohort

**DOI:** 10.1038/s41598-023-35298-y

**Published:** 2023-06-10

**Authors:** Luísa Castro, Luísa Brant, Maria de Fátima Diniz, Paulo Lotufo, Isabela Judith Bensenor, Dora Chor, Rosane Griep, Sandhi Maria Barreto, Antonio Luiz Ribeiro

**Affiliations:** 1 Longitudinal Study of Adult Health - ELSA Brasil, Federal University of Minas Gerais, Prof. Alfredo Balena, 110 - Santa Efigênia, Belo Horizonte, MG 30130-100 Brazil; 2 Center for Clinical and Epidemiological Research, Hospital Universitário, Universidade de São Paulo, São Paulo, Brazil; 3 National School of Public Health, Oswaldo Cruz Foundation, Rio de Janeiro, Brazil; 4 Laboratory of Education in Environment and Health, Oswaldo Cruz Foundation, Rio de Janeiro, Brazil

**Keywords:** Risk factors, Hypertension, Diabetes

## Abstract

Insulin resistance (IR) is defined as the subnormal response to insulin action on its target tissues. Studies suggest that IR may increase the risk of hypertension, but the results are inconsistent and it is not known whether such an effect is independent of overweight/obesity. We aimed to evaluate the association between IR and the incidence of prehypertension and hypertension in the Brazilian population and whether this association is independent of overweight/obesity. In 4717 participants of the Brazilian Longitudinal Study of Adult’s Health (ELSA-Brasil), free of diabetes and cardiovascular disease at baseline (2008–2010), we investigated the incidence of prehypertension and hypertension after a mean follow-up of 3.8 ± 0.5 years. Insulin resistance at baseline was assessed by the Homeostasis Model Assessment of Insulin Resistance (HOMA-IR) index, defined if above the 75th percentile. The risk of IR-associated prehypertension/hypertension was estimated by multinomial logistic regression after adjustment for confounding factors. Secondary analysis were stratified by body mass index. The mean (SD) age of participants was 48 (8) years, 67% were women. The 75th percentile of HOMA-IR at baseline was 2.85. The presence of IR increased the chance of developing prehypertension by 51% (95% CI 1.28–1.79) and hypertension by 150% (95% CI 1.48–4.23). In individuals with BMI < 25 kg/m^2^, the presence of IR remained associated with the incidence of prehypertension (OR 1.41; 95% CI 1.01–1.98) and hypertension (OR 3.15; 95% CI 1.27–7.81). In conclusion, our results suggest that IR is a risk factor for hypertension, regardless of the presence of overweight or obesity.

## Introduction

Insulin resistance (IR) is defined as a subnormal response to the action of insulin on its target tissues, primarily liver, muscle, and adipose tissue^1^. The term encompasses a broad continuum, ranging from individuals who maintain normal glucose homeostasis due to increased endogenous insulin production and do not yet have diabetes, to individuals with diabetes who require exogenous insulin to maintain glucose homeostasis^1^. There is no absolute value in the literature established as a cutoff point for the HOMA index to define IR, since it may vary according to each population studied and race^2^. Therefore, in general, HOMA percentile values within a given population are considered to classify individuals in relation to IR. The value suggested by the World Health Organization is above the 75th percentile^3^.

Diabetes is a known predictor of hypertension^4^, but the association between IR and the incidence of hypertension in non-diabetic individuals is controversial^2,3,5^, as is the role of obesity in the relationship between IR and the incidence of hypertension. While some longitudinal studies showed that IR is a risk factor for hypertension independent of body mass index (BMI)^2,3,6,7^, other studies found no association between IR and incident hypertension after adjustments for BMI or showed that IR did not increase the chance of hypertension in individuals with normal BMI, and lastly that the incidence of hypertension in overweight/obese groups occurred independently of the presence of IR^5,8^.

As such, we aimed to evaluate the association between IR and the incidence of prehypertension and hypertension in Brazilian adults and whether this association is independent of overweight/obesity in participants of the ELSA-Brasil cohort free of diabetes and CVD. Prehypertension was included as an outcome because it not only increases the risk of developing hypertension over the next 3 years by 20–40%, but it relates to cardiovascular events^9,10^. Importantly, although a recent publication of the Hispanic Community Health Study/Study of Latinos showed, in a cross-sectional analysis, that IR was directly correlated with systolic and diastolic blood pressure, even in individuals without diabetes^11^, we did not identify multicenter cohorts carried out in Latin American population that have studied the impact of IR in individuals without diabetes on the development of prehypertension and hypertension.

## Methods

### Participants

This study analyzes data from the ELSA-Brasil study, a multicenter Brazilian cohort of civil servants aged 35–74 years of six research centers in Brazil (the Federal Universities of Minas Gerais, Rio Grande do Sul, Bahia, and Espírito Santo; the University of São Paulo; and the Oswaldo Cruz Foundation in Rio de Janeiro), aiming at identifying determinants of cardiovascular disease (CVD) and diabetes^12,13^. Data collection for the 15,105 ELSA-Brasil participants started in 2008 and occurred through visits of participants to the research centers, where interviews about sociodemographics, lifestyle and medical history, anthropometrics, biochemical tests, and imaging tests were performed^12^.

To date, three visits of participants have been conducted, with the data herein analyzed analysis being from the first (2008 to 2010) and second assessment (2012 to 2014). More details are available about the design and cohort profile of ELSA-Brazilian’s previous publications^14–19^. Briefly, the mean age of the entire cohort was 52 years, 55% were female, 52% identified as white, and 44% as brown or black. Most (53%) had higher education, due to the nature of the cohort being employees of research institutions, 36% had hypertension, 23% were obese, and 20% had diabetes^2^.

**Figure 1:**
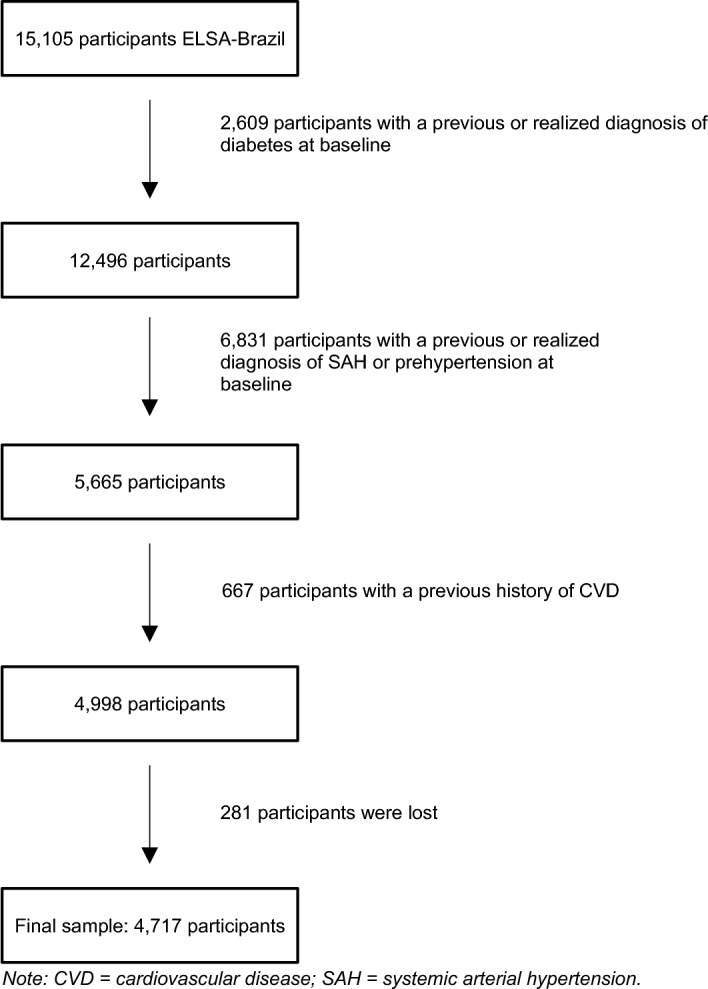
Flowchart of participants. Diagram depicting eligible sample.

In the present study, participants with valid fasting glucose, oral glucose tolerance test (OGTT), insulin, and glycated hemoglobin tests at baseline were included, and participants with the following characteristics at baseline were excluded*:* (a) prevalent diabetes(n = 2609); (b) prevalent hypertension or prehypertension (n = 6831); (c) those who were lost to follow-up at the second study assessment for any reason, including death (n = 281); (d) prevalent CVD (n = 667), defined as self-reported history of heart failure, coronary artery disease or revascularization, or previously defined major electrocardiographic abnormalities^20^. The remaining 4717 participants were included in the analyses (Fig. 1) and the mean follow-up was 3.8 ± 0.5 years.

ELSA-Brasil procedures were approved by the National Research Ethics Committee (CONEP/MS 976/2006) and by Research Ethics Committees of Universidade Federal da Bahia, Universidade Federal de Minas Gerais, Universidade Federal do Espírito Santo, Fundação Oswaldo Cruz, Universidade de São Paulo e Universidade Federal do Rio Grande do Sul. All methods were performed in accordance with the relevant guidelines and regulations. Informed consent was obtained from all subjects.

### Glucose, insulin, insulin resistance, and diabetes

Fasting blood glucose and insulinemia were measured after a 12-h fast. Blood glucose was measured by the Hexokinase method (enzymatic) in the ADVIA 1200 Siemens® (Deerfield, Illinois) equipment, and insulinemia by enzyme-linked immunosorbent assay (ELISA), Centaur Siemens® equipment. For TOTG, blood glucose was measured 2 h after the ingestion of a solution containing 75 g of glucose^17,27^. The IR was measured by HOMA-IR, calculated by the formula: insulin (μU/mL) × [glucose (mmol/L)/22.5]. Values above the 75th percentile were considered IR^30–32^.

Although the gold standard for IR quantification is the euglycemic-hyperinsulinemic clamp technique, it is costly, invasive, and difficult to perform^21,22^. As such, as in other epidemiological studies, indirect methods are used, the Homeostasis Model Assessment of Insulin Resistance (HOMA) index and the Quantitative insulin sensitivity check index (QUICKI) the most commonly used^23^. In this study, HOMA-IR was chosen because it is a method validated through the euglycemic-hyperinsulinemic clamp technique^22,24^.

Diabetes was defined as a self-reported medical diagnosis of diabetes during the interview, use of antidiabetic medications at the time of entering the study, fasting glucose ≥ 126 mg/dl and/or 2 h post-TOTG glucose ≥ 200 mg/dl and/or glycohemoglobin ≥ 6.5% at baseline tests^12,17^.

### Hypertension

Blood pressure was measured by three measurements 1 min apart from each other and after 5 min of rest obtained with an oscillometric sphygmomanometer (Omron 765CP; Omron, Kyoto, Japan). The mean of the two last measurements was considered^17^. At baseline, prevalent hypertension was defined as systolic blood pressure (SBP) ≥ 140 mmHg or diastolic blood pressure (DBP) ≥ 90 mmHg or use of antihypertensive medications at baseline, and prevalent prehypertension was defined as SBP values of 120–139 mmHg and DBP values of 80–89 mmHg, in individuals without antihypertensive medication use^17^. The outcomes of incident hypertension and prehypertension were defined as SBP ≥ 140 mmHg or DBP ≥ 90 mmHg at the second visit or if participants had started antihypertensive use during the period between the first and second visit^14^. Prehypertension was defined as SBP values of 120–139 mmHg or DBP values of 80–89 mmHg on the second visit, in individuals without antihypertensive medication use. Self-report was not considered a diagnostic criteria^14,17,19^.

### Covariates

Social and demographic data, medication use, smoking, alcohol use, and degree of physical activity were collected through interviews during the baseline visit. Race/skin color was self-reported according to the Brazilian Demographic Census (white, brown, black, yellow—traditionally used in the country to classify Asian individuals, and indigenous). This variable was used in 3 categories for the multivariable model—black, brown, and white—due to the small number of responses for yellow and indigenous (less than 3%). Education level was classified in two categories: complete high school or higher, and incomplete high school or lower. Participants who reported having smoked at least 100 cigarettes or five packs in their lifetime and still smoked were considered smokers; those who no longer smoked at the time of the interview were classified as former smokers; the remainder were considered nonsmokers. Excessive alcohol use was defined as consumption of > 210 g of alcohol/week for males and > 140 g of alcohol/week for females^13^. Physical activity was measured by the dimension of leisure-time physical activity using the International Physical Activity Questionnaire (IPAQ), a *long modified version* of^25^, validated in Brazil and was analyzed stratified into categories of low, moderate, or high level physical activity.

Anthropometric parameters were measured using standardized techniques such as weight (kg), height (m), and waist circumference (cm). The latter was performed in fasting, after emptying the bladder with an anthropometric tape, from the midpoint between the upper margin of the iliac crest and the last rib, in the middle axillary line, with the participant in the upright and relaxed posture^17^. BMI was calculated as weight (in kg) divided by height (in meters squared)^17^. Obesity was defined as BMI ≥ 30 kg/m^2^, overweight as BMI ≥ 25 and < 30 kg/m^2^. Abdominal obesity was defined as an abdominal circumference ≥ 102 cm in men and ≥ 88 cm in women. Self-report was not considered a diagnostic criteria^17^.

### Statistical analysis

Descriptive analyses were presented as mean (standard deviation) or median (interquartile range). Categorical variables were described as frequencies and percentages. Group comparisons were made using Kruskal–Wallis or ANOVA tests for continuous variables and the Chi-square test for categorical variables. For incidence of prehypertension and hypertension, IR at baseline was considered as the exposure factor and incident prehypertension and hypertension at the second assessment of ELSA-Brasil was considered the outcome.

To investigate the association of IR and incidence of prehypertension and hypertension, univariable multinomial logistic regressions were first performed, and then sequential multivariable multinomial logistic regression models were analyzed, with adjustment variables measured at baseline: Model 1—crude model; Model 2—adjusted for sex, age, race/skin color, and education; Model 3—adjusted for sex, age, race/color, education, alcohol use, kidney function, smoking, heart rate, and physical activity. The aforementioned variables were included in the multivariable models since they were considered confounding factors for the association of IR and the outcomes^26^.

We did not adjust by baseline pressure values because we already excluded all those with higher baseline pressure levels (with prehypertension) from our sample. We understand that this strategy greatly reduces the effect of eventually higher basal pressure levels.

Analysis was performed to verify the presence of interaction between IR and overweight/obesity in the incidence of outcomes. Subsequently, to investigate whether the association of IR with prehypertension and hypertension occurs independently of overweight/obesity, a secondary analysis stratified by BMI was performed (BMI < 25 kg/m^2^ was considered as absence and overweight/obesity)^5^, with the same other covariates used for adjustments.

The results were presented as Odds Ratio (OR) considering a 95% confidence interval (CI). SPSS Statistics for Macintosh, Version 22.0 software (IBM Corp. Armonk, NY, 2013).

## Results

Sociodemographic data and clinical characteristics collected at baseline are shown in Supplement 1. Participants had a mean (SD) age of 48 (7.9) years, 67% were women and 63% had higher education.

The median (IIq) value of HOMA-IR was 1.97 (1.37–2.85) and IR was present in 1179 participants (HOMA-IR > 2.85). More than 95% of the patients in our sample had creatinine clearance above 60 mL/min, thirty six percent of the participants were overweight and 11% obese (data not shown).

Table 1 shows participants’ characteristics stratified by the presence of incident prehypertension or hypertension.

**Table 1:** Sociodemographic data and clinical characteristics at baseline of the 4717 participants included free of diabetes, prehypertension, hypertension and cardiovascular disease, categorized by incident normotension, prehypertension or hypertension—ELSA-Brasil 2008–2014.

Characteristic	Absence of incident prehypertension/hypertension	Incident prehypertension	Incident hypertension
Sex n (%)			
Male	1099 (70.7)	435 (27.7)	36 (2.3)
Female	2498 (79.4)	610 (19.4)	39 (1.2)
Average age (SD)	47 (7.8)	49 (8.1)	50 (9.3)
Color n (%)			
Black	332 (10.2)	136 (14.2)	13 (20.3)
Brown	840 (25.8)	266 (27.8)	15 (23.4)
White	1982 (60.8)	524 (58.4)	34 (53.1)
Yellow	86 (2.6)	21 (2.2)	2 (3.1)
Indigenous	21 (0.6)	9 (0.9)	0 (0.0)
Incomplete high school or lower n (%)	1222 (70.8)	475 (27.5)	30 (1.7)
Complete high school or higher n (%)	2375 (79.4)	570 (19.1)	45 (1.5)
Presence of overweight/obesity	1561 (69.5)	635 (28.3)	49 (2.2)
Absence of overweight/obesity	2032 (82.4)	407 (16.5)	26 (1.1)
Presence of IR	2943 (77.9)	330 (28,0)	33 (1,2)
Absence of IR	816 (69.2)	715 (20.2)	42 (2.8)

*Hypertension* Hypertension, *IR* Insulin resistance.

*297 people did not inform race/color.

### Incident prehypertension and hypertension

The incidence of prehypertension was 22.2% (n = 1045) and of hypertension was 1.6% (n = 75). In univariable multinomial logistic regression analyses, the risk factors for prehypertension and hypertension were: male sex, age, black race/skin color, abdominal obesity, obesity, and IR (Table 2). Education less than high school education and brown race/skin color were risk factors only for prehypertension (Table 2).

**Table 2:** Univariable multinomial models involving the 4717 included participants free of diabetes and cardiovascular disease, categorized by incident normotension, prehypertension, or hypertension—ELSA-Brasil 2008–2014.

Characteristic	Prehypertension OR [95% CI]	Hypertension OR [95% CI]
Male sex	1.62 [1.41–1.87]	2.10 [1.33–3.32]
Age in years	1.03 [1.02–1.03]	1.04 [1.01–1.10]
Black color*	1.55 [1.24–1.93]	2.28 [1.19–4.37]
Brow color	1.20 [1.01–1.42]	1.04 [0.56–1.92]
Education below complete high school education n (%)	1.62 [1.41–1.86]	1.30 [0.81–2.10]
IR	1.57 [1.35–1.83]	2.68 [1.69–4.25]
Abdominal obesity	1.61 [1.38–1.89]	2.70 [1.69–4.32]

*Hypertension* Hypertension, *OR* Odds ratio, *IR* Insulin resistance.

*Yellow or indigenous race/color were not evaluated due to the small number of individuals.

**Figure 2:**
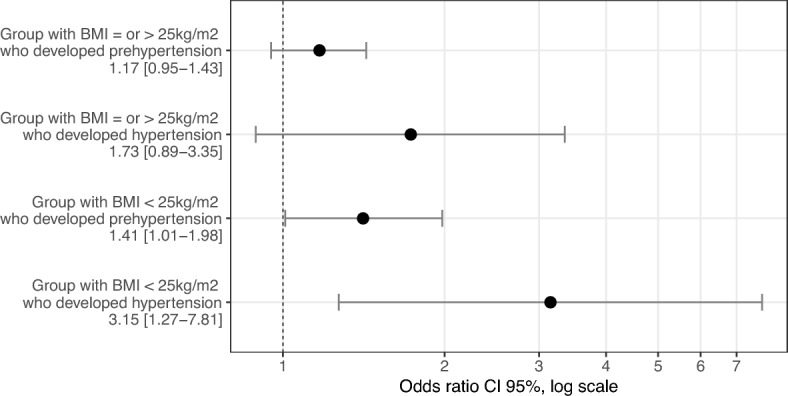
Outcomes plot. Impact of insulin resistance in the incidence of prehypertension and hypertension stratified by body mass index.

Multivariable multinomial logistic regression models are shown in Table 3: insulin resistance was a predictor of prehypertension and hypertension in model 1 (no adjusted) and in model 2 (adjusted by sex, age, ethnicity and educational level). In the final model (model 3 – in which traditional cardiovascular risk factors were included as covariates), IR remained a strong predictor of prehypertension and hypertension incidence (OR 1.51; 95% CI 1.28–1.79 and OR 2.50, 95% CI 1.48–4.23 respectively), with greater strength of association for hypertension. The interaction analysis between IR and overweight/obesity was significant (*p* = 0.034), so further analyses stratified by the presence of overweight/obesity were performed. In the group without overweight or obesity (n = 2469), IR increased the chance of prehypertension by 41% (95% CI 1.01–1.98) and of hypertension by 215% (95% CI 1.27–7.81). In the overweight/obese group (n = 2248), IR had no significant association with prehypertension or hypertension (Fig. 2).

**Table 3:** Incidence of prehypertension and hypertension according to the presence of insulin resistance and multivariable logistic regression models in 4717 participants included free of diabetes, and cardiovascular disease—ELSA-Brasil 2008–2014.

Model	Prehypertension	Hypertension
	OR (CI 95%)	OR (CI 95%)
Model 1 (insulin resistance)	1.57 (1.35–1.83)	2.68 (1.69–4.25)
Model 2 (model 1 adjusted by sex, age, ethnicity and educational level)	1.52 (1.29–1.79)	2.24 (1.35–3.73)
Model 3 (model 2 + traditional risk factors for pre-hypertension/hypertension**)	1.51 (1.28–1.79)	2.50 (1.48–4.23)

139 people self-reported yellow or indigenous were not included due to low numbers; 297 people did not report their color.

*OR* Odds ratio, *CI* Confidence interval.

**Alcohol use, kidney function, heart rate, smoking and physical activity.

## Discussion

In this study, including 4717 Brazilian adults free of diabetes and CVD, we showed that IR increased the risk of developing prehypertension by 51% and hypertension by 150% after 3.8 ± 0.5 years of follow-up. Importantly, IR was able to predict the incidence of prehypertension and hypertension in individuals with normal BMI, revealing that it is a risk factor independent of overweight or obesity.

Some previous studies have shown an association of IR with increased risk of hypertension^2,3,7^, while others found no such association^3,5,8^. This study is the first multicenter prospective study to assess the impact of IR on the development of prehypertension and hypertension in individuals without diabetes in Latin America. Prehypertension increases the risk for developing hypertension by 20–40% in 3 years, with results varying between populations studied and age of onset of hypertension (higher risk among younger people)^27^. In addition, prehypertension has been shown to increase the risk of cardiovascular events. Vasan et al. for example, demonstrated that men and women with prehypertension had a 1.6 and 2.5-fold increased chance, respectively, of developing a cardiovascular event in 10 years when compared to normotensive individuals^28^. Hsia et al. demonstrated a 66% increased risk of cardiovascular events in postmenopausal women with prehypertension (over a period of 7.7 years), compared with normotensive women^10^. Given the importance of prehypertension as an independent cardiovascular risk factor, in this study we considered prehypertension as an outcome, and not just hypertension.

The North American Multi-Ethnic Study of Atherosclerosis (MESA) cohort, showed that HOMA-IR above the 75th percentile (> 1.7) was a predictor for hypertension (n = 3513) (RR 1.44; 95% CI 1.16–1.80), however, when the secondary analysis was performed excluding those with prehypertension at baseline, impaired glucose tolerance did not increase the chance of developing hypertension^29^. Our data, in contrast, showed an increased risk for hypertension even when only normotensive individuals were included at baseline, suggesting that IR contributes to blood pressure elevation across the continuum and not only in those at higher risk of becoming hypertensive because they already have higher baseline blood pressure levels.

Previous data had already suggested that there might be an interaction between BMI and IR regarding their ability to predict hypertension. Lytsi and colleagues (n = 1846) demonstrated that individuals with IR and normal BMI did not have a higher risk of hypertension, while those with IR associated with overweight/obesity had a higher risk of progression to hypertension^5^. Similarly, a Greek study (n = 141) showed a 98% increased chance of hypertension in women with IR, however, this association also lost significance when the model was adjusted for the presence of obesity^8^. In the present study, when our models were performed with individuals above the ideal BMI, IR ceased to have a significant association with the outcomes, suggesting that in overweight/obese individuals, IR alone would have no additional impact on the development of prehypertension or hypertension. However, it is important to note that our data showed an increased risk for prehypertension and hypertension when the final model was performed in a population free of overweight or obesity, suggesting that IR predicts increased blood pressure levels independently of BMI in this population. This finding suggests that IR may be an earlier marker than overweight/obesity for hypertension prediction^30^.

The IR and compensatory hyperinsulinemia contribute to the pathophysiology of hypertension in a multifactorial manner. IR has been shown to be related to increased peripheral arterial resistance and greater arterial stiffness^31^. Moreover, some studies indicate that insulin has a peripheral vasodilator effect by stimulating the production of endothelial nitric oxide and by reducing the intracellular concentration of calcium in muscle cells of the arterial wall^32^. Therefore, an individual with resistance to the action of insulin would have less vasodilator action of the hormone. Moreover, insulin may have an anti-natriuretic action in the distal renal tubules and increase the activation of the renin–angiotensin–aldosterone system^2^. Consequently, individuals with compensatory hyperinsulinemia in the context of IR may have an exacerbation of these effects, which would lead to greater vasoconstriction and increased total circulating plasma volume^2^, both of which are important in the pathophysiology of hypertension.

Our study also innovates by evaluating the impact of IR on prehypertension and hypertension in people without diabetes in Latin America. Another differential in our study is the large number of individuals included, the comprehensiveness of the data collected, which allowed the evaluation of potential confounding factors, and the socio-demographic diversity inherent of the Brazilian population. This factor is essential in studies that assess the incidence of hypertension, since it is clear in the literature the importance of sociodemographic factors, such as race and socioeconomic status, in the development of CVD and its risk factors^33,34^.

As a limitation of the study, we can cite the loss of follow-up of some individuals. However, besides being very small (5.6%), the losses in this study were not associated with risk factors for hypertension, such as age, sex, HOMA-IR, color, education, and the presence of obesity. Another limitation is that ELSA-Brasil, although composed of citizens living in six Brazilian cities, does not include all the spectrum of the Brazilian population, such as the very poor or unemployed. Furthermore, our sample was predominantly made up of women, white people and individuals with higher education, and as such this need to be taken into account when considering generalizability^35^. A limitation that applies to all studies on IR and hypertension is the fact that there is no HOMA-IR cutoff value for defining IR, being it defined by percentiles that inevitably vary according to the population studied^36^.

Lastly, as perspectives, due to the importance of IR for the development of prehypertension/hypertension, IR might be an interesting tool to consider by healthcare teams; and treatment of IR should be advocated with the promotion of healthy behaviors or even medication, for specific situations^1^.

In conclusion, IR acted as a risk factor for the development of hypertension and pre-hypertension in Brazilian adults free of diabetes, and without obesity/overweight. As the ELSA-Brasil follow-up time advances, it will be possible to further investigate how IR affects the acceleration in SBP and DBP over the years.

## Supplementary Information

Supplementary Information.

## Data Availability

The datasets used and/or analysed during the current study available from the corresponding author on reasonable request.
