# Supplementary material for: Association of hypertension and insulin resistance in individuals free of diabetes in the ELSA-Brasil cohort
Source: Sci Rep. 2023 Jun 10;13:9456. doi: 10.1038/s41598-023-35298-y (PMC10257662; doi:10.1038/s41598-023-35298-y)
Supplement: Supplementary file 1 — Supplementary Information. [file 41598_2023_35298_MOESM1_ESM.docx]

**Supplement 1.** Clinical-laboratory characteristics at baseline of the 4,717 included participants free of diabetes and cardiovascular disease categorized by presence or absence of insulin resistance - ELSA-Brasil 2008-2014.

| **Characteristic** | **Presence of IR** | **Absence of IR** | **p** |
| --- | --- | --- | --- |
| Sex n (%)  male (33%)  female (67%) | 412(26.2)  767(24.4) | 1,158(73.8)  2,380(75.6) | 0.06 |
| Age (mean [SD]) | 49(8.1) | 48(7.8) | 0.10 |
| Education – complete high school or higher n (%) | 716(23.9) | 2,274(76.1) | 0.20 |
| Color n (%)  Black  Brown  White  Yellow  Indigenous | 123(25.6)  291(26.0)  623(24.5)  22(20.2)  7(23.3) | 358(74.4)  830(74.0)  1,917(75.5)  87(79.8)  23(76.7) | 0.37 |
| Presence of abdominal obesity n (%) | 509(48.9) | 531(51.1) | < 0,001 |
| Creatinine Clearance >60mL/min  n(%) | 1,152(98.0) | 3,499(99.0) | 0.01 |

*Note: IR = insulin resistance*
